# Voltage-Gated Ion Channel Dysfunction Precedes Cardiomyopathy Development in the Dystrophic Heart

**DOI:** 10.1371/journal.pone.0020300

**Published:** 2011-05-23

**Authors:** Xaver Koenig, Sandra Dysek, Stefanie Kimbacher, Agnes K. Mike, Rene Cervenka, Peter Lukacs, Katrin Nagl, Xuan B. Dang, Hannes Todt, Reginald E. Bittner, Karlheinz Hilber

**Affiliations:** 1 Center for Physiology and Pharmacology, Department of Neurophysiology and Pharmacology, Medical University of Vienna, Vienna, Austria; 2 Center for Anatomy and Cell Biology, Neuromuscular Research Department, Medical University of Vienna, Vienna, Austria; Brigham & Women's Hospital - Harvard Medical School, United States of America

## Abstract

**Background:**

Duchenne muscular dystrophy (DMD), caused by mutations in the dystrophin gene, is associated with severe cardiac complications including cardiomyopathy and cardiac arrhythmias. Recent research suggests that impaired voltage-gated ion channels in dystrophic cardiomyocytes accompany cardiac pathology. It is, however, unknown if the ion channel defects are primary effects of dystrophic gene mutations, or secondary effects of the developing cardiac pathology.

**Methodology/Principal Findings:**

To address this question, we first investigated sodium channel impairments in cardiomyocytes derived from dystrophic neonatal mice prior to cardiomyopahty development, by using the whole cell patch clamp technique. Besides the most common model for DMD, the dystrophin-deficient mdx mouse, we also used mice additionally carrying an utrophin mutation. In neonatal cardiomyocytes, dystrophin-deficiency generated a 25% reduction in sodium current density. In addition, extra utrophin-deficiency significantly altered sodium channel gating parameters. Moreover, also calcium channel inactivation was considerably reduced in dystrophic neonatal cardiomyocytes, suggesting that ion channel abnormalities are universal primary effects of dystrophic gene mutations. To assess developmental changes, we also studied sodium channel impairments in cardiomyocytes derived from dystrophic adult mice, and compared them with the respective abnormalities in dystrophic neonatal cells. Here, we found a much stronger sodium current reduction in adult cardiomyocytes. The described sodium channel impairments slowed the upstroke of the action potential in adult cardiomyocytes, and only in dystrophic adult mice, the QRS interval of the electrocardiogram was prolonged.

**Conclusions/Significance:**

Ion channel impairments precede pathology development in the dystrophic heart, and may thus be considered potential cardiomyopathy triggers.

## Introduction

Duchenne muscular dystrophy (DMD) is the most common and devastating form among the human muscular dystrophies. The disease is characterised by progressive muscle weakness with cycles of muscle necrosis and regeneration. The gene defect for DMD was mapped to an X chromosome gene that encodes the intracellular protein dystrophin. This protein seems to act as a linker between the cytoskeleton and the extracellular matrix through its interaction with proteins of the dystrophin-associated protein complex (DAPC) [1]. Besides the relatively well described skeletal muscle degenerative processes, DMD and some other muscular dystrophy types are also associated with cardiac complications, including cardiomyopathy and cardiac arrhythmias [2], [3]. These contribute significantly to the morbidity and mortality observed [2], [4], and, considering the increased life span of dystrophy patients nowadays, have become a crucial issue [4], [5]. The current understanding of the mechanisms generating cardiac complications, however, is limited.

In recent years it has become apparent that ion channels are part of large multi-protein complexes [6]. Disruption of any protein member of such a particular “ion channel complex” has the potential to affect the function and localisation of the associated channels, and consequently, may alter cellular excitability. Recent research has exposed that also protein members of the DAPC interact with voltage-gated ion channels. For example, the main cardiac sodium channel isoform Na_v_1.5 interacts with dystrophin [7] and syntrophins [7], [8], and these interactions modulate the channel. Accordingly, disruption of the DAPC in dystrophin-deficient cardiomyocytes from the mdx mouse (most commonly used mouse model for human DMD with a mutation in the dystrophin gene [9]) induces reduced Na_v_1.5 protein levels and sodium current densities [5], [7], [10]. Importantly, decreased sodium current may contribute to the impairment in cardiac electrical conduction observed in DMD patients (e.g. [11], [12]).

Lately, transgenic mice with reduced expression of Na_v_1.5 channels in the heart were shown to develop a dilated cardiomyopathy [13]. The authors demonstrated that the greater the reduction in sodium current was, the faster was also the onset and progression of the cardiomyopathy. These findings, together with reports associating mutations in the SCN5A gene (coding for the cardiac Na_v_1.5 channel) with cardiomyopathy (e.g. [14], [15]), strongly suggest that aberrant Na_v_1.5 channel expression and function should be considered as potential underlying mechanisms for cardiomyopathy. Since cardiomyopathy development is also an important feature of the pathology observed in DMD patients [2], [3], as well as in DMD mouse models (e.g. [16]–[18]), reduced Na_v_1.5 currents and/or impaired Na_v_1.5 channel gating in dystrophic cardiomyocytes [7], [10] may be causally involved in the formation of dystrophic cardiomyopathy. If so, sodium channel impairments should precede cardiomyopathy development in the dystrophic heart, which has not been demonstrated as yet.

To test the *hypothesis* that ion channel abnormalities occur prior to cardiac pathology, in this study, we investigated the functional properties of both sodium and calcium channels in cardiomyocytes derived from the ventricles of dystrophic neonatal mouse hearts. Besides the dystrophin-deficient mdx mouse, we also used mice additionally carrying a mutation in the utrophin gene [16], [19]. The latter mouse model (mdx-utr) more closely resembles the general pathology observed in DMD patients, and develops a more severe cardiomyopathy with an earlier onset compared to mdx (e.g. [16], [20]). Because both young adult mdx mice (e.g. [16], [18], [21]–[23]), as well as mdx-utr double mutant mice of less than seven weeks of age [16] lack any signs of pathological cardiac morphology or impaired heart function, ion channel defects observed in dystrophic neonatal cardiomyocytes can be considered primary effects of dystrophic gene mutations preceding cardiomyopathy development. Here we report that ion channel abnormalities are already present in cardiomyocytes of the dystrophic neonatal heart.

## Results

### Sodium current properties in normal and dystrophic neonatal cardiomyocytes

**Figure 1:**
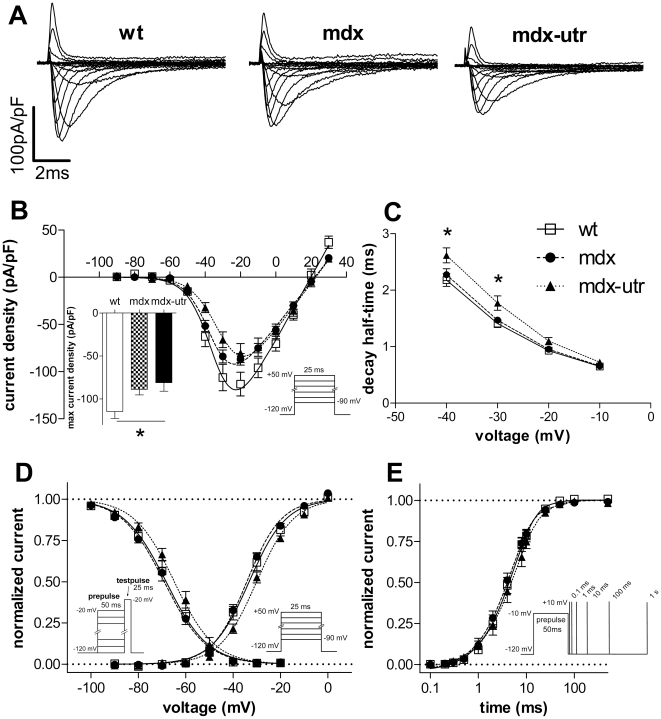
Sodium channel properties in wt and dystrophic neonatal cardiomyocytes. **A.** Original current traces of a typical wt, mdx, and mdx-utr cardiomyocyte, elicited by depolarising voltage-steps. **B.** Current-voltage relationships derived from a series of experiments as shown in A. The Inset (bar graph) shows a comparison of the maximum current densities between wt and dystrophic cardiomyocytes. **C.** Comparison of the current decay kinetics between wt and dystrophic cardiomyocytes at different membrane voltages. Decay half-times represent the time periods between the current peak and the time point at which the current had decayed to 50%. * indicates that ANOVA revealed a significant difference (p<0.05) between the respective parameters of the three tested groups. Tukey's Post Hoc test for comparison between two groups revealed significant differences (p<0.05) between mdx-utr and wt, and between mdx-utr and mdx. **D.** Voltage-dependencies of activation and steady-state inactivation in wt and dystrophic cardiomyocytes. **E.** Recovery from inactivation in wt and dystrophic cardiomyocytes. Data were fit with a single exponential function. The pulse protocols are shown as insets in the respective graphs. Data points represent means ± SEM. Detailed statistics and n-values are given in Table 1.

Figure 1 shows typical original traces of sodium currents (A) elicited by various depolarizing voltage steps of a wt, mdx, and mdx-utr cardiomyocyte. In addition, a summary of the current-voltage relationships (B), derived from a series of such experiments, is shown. It can be observed that the current densities of dystrophic cardiomyocytes were significantly decreased (also see Table 1). This suggests that dystrophin-deficiency reduces the number of functional sodium channels. Extra utrophin-deficiency, on the other hand, did not produce a considerable additional effect in neonatal cardiomyocytes.

**Table 1:** Sodium and barium current parameters in wt and dystrophic cardiomyocytes.

		Activation					Inactivation		
		V05	k	Vrev	Current Density	n	V05	k	n
**Na^**+**^ currents**		mV	mV	mV	pA/pF		mV	mV	
**Neonatal**	**wt**	*−34.0±1.6*	6.6±0.3	20.0±1.4	*−115±8*	(11)	*−70±2.5*	*7.8±0.2*	(8)
	**mdx**	*−34.2±0.8*	6.7±0.3	22.4±0.6	*−89±6* &	(15)	*−68.4±1.2*	*8.7±0.2* &	(15)
	**mdx-utr**	*−29.5±1.2* § $	6.8±0.2	22.4±0.8	*−81±10* §	(12)	*−63.7±1.7*	*7.9±0.2* $	(15)
**Adult**	**wt**	−27.6±1.0	6.1±0.1	**23.5±0.9**	**−201±25**	(11)	−56.7±0.9	8.4±0.1	(10)
	**mdx**	−27.3±1.1	6.2±0.3	**24.7±0.9**	**−122±17** &	(11)	−58.2±1.3	8.3±0.3	(11)
	**mdx-utr**	−27.4±1.3	6.7±0.3	**20.3±0.9** $$	**−69±9** §§§ $	(10)	−56.7±0.9	8.1±0.2	(10)
**Ba^2**+**^ currents**									
**Neonatal**	**wt**	6.6±1.5	7.5±0.3	60.6±1.3	−7.1±1.0	(15)	−22.8±0.6	7.3±1.1	(6)
	**mdx**	5.9±0.9	6.8±0.3	59.1±1.3	−5.9±1.0	(13)	−19.9±1.5	7.6±0.7	(5)
	**mdx-utr**	7.1±1.3	7.6±0.4	60.7±1.3	−5.8±0.8	(14)	−21.5±1.3	7.1±0.6	(6)

The given parameters were obtained by the analysing procedures described in Materials and Methods. V_0.5_ is the voltage at which half-maximum activation or inactivation occurred, and k represents the slope factor. V_rev_ is the reversal potential, and n gives the number of experiments. Values represent means ± SEM. Data in italic indicate that ANOVA revealed a significant difference (p<0.05) between the respective parameters of the three different groups (wt, mdx, and mdx-utr). Data in bold indicate that ANOVA revealed p<0.01. Statistical comparisons between two groups were made using Tukey's Post Hoc test. Here,

& p<0.05 indicates a significant difference between mdx and wt.

§ p<0.05 and

§§§ p<0.001 indicate significant differences between mdx-utr and wt.

$ p<0.05 and

$$ p<0.01 indicate significant differences between mdx-utr and mdx.

Analysis of the current decay after channel activation (Figure 1C) revealed that the kinetics of inactivation was similar in wt and mdx cardiomyocytes, but, over a certain voltage range, significantly slowed in mdx-utr cells. These data suggest that extra utrophin-deficiency reduces inactivation.

Comparison of the sodium channel activation and steady-state inactivation curves between wt and dystrophic cardiomyocytes (Figure 1D) reveals that dystrophin-deficiency alone (mdx) does not generate any effect. In contrast, extra utrophin-deficiency moderately shifts both curves to the right. This is represented by a significant shift of V_0.5_, the voltage at which half-maximum activation or inactivation occurred, to more positive values in mdx-utr cardiomyocytes (Table 1). These results suggest that extra utrophin-deficiency affects the voltage-dependence of gating in neonatal cardiomyocytes.

Finally, Figure 1E shows that the recovery from inactivation is similar in wt and dystrophic neonatal cardiomyocytes.

### Properties of currents through L-type calcium channels in normal and dystrophic neonatal cardiomyocytes

**Figure 2:**
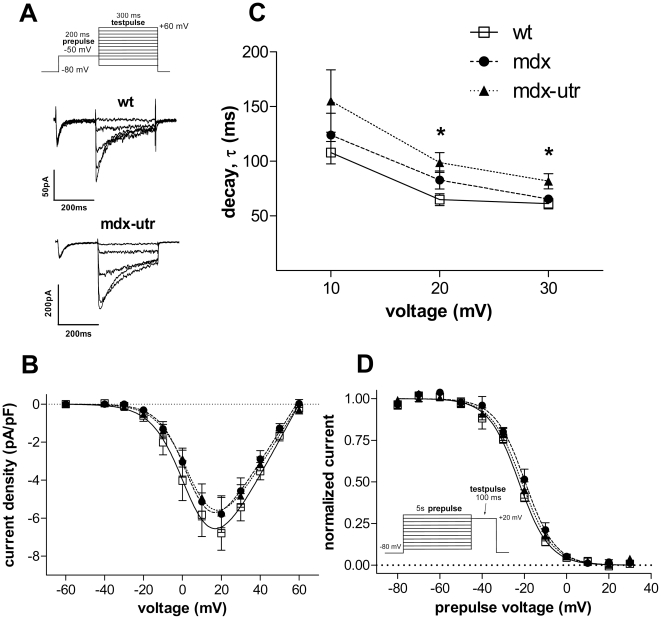
Calcium channel properties in wt and dystrophic neonatal cardiomyocytes. **A.** Original barium current traces (five episodes displayed) of a wt and mdx-utr cardiomyocyte, elicited by the pulse protocol on top. The prepulse was used to eliminate currents through T-type calcium channels. **B.** Current-voltage relationships derived from a series of experiments on wt and dystrophic cardiomyocytes as shown in A. **C.** Comparison of the current decay kinetics between wt and dystrophic cardiomyocytes at various membrane voltages. τ –values were derived from single exponential fits of the current decay after channel activation. * indicates a significant difference (ANOVA, p<0.05) between the three tested groups. Tukey's Post Hoc test revealed a significant difference (p<0.05) between mdx-utr and wt, but not between mdx-utr and mdx. **D.** Voltage-dependency of steady-state inactivation in wt and dystrophic cardiomyocytes. For n-values see Table 1.

Having shown that sodium channels are impaired already in dystrophic neonatal cardiomyocytes (prior to cardiomyopathy development), it was of interest to test if this is a special feature of sodium channels in the dystrophic neonatal heart, or more generally also applies to other ion channels. Therefore, we compared the properties of calcium channels in wt and dystrophic neonatal cardiomyocytes. Figure 2 shows that the barium current densities were similar in wt and dystrophic cardiomyocytes (B). Analysis of the current decay after channel activation revealed that the kinetics of inactivation was slowed in dystrophic cells (Figure 2C). This effect was somewhat more pronounced in mdx-utr compared to mdx cardiomyocytes, suggesting that extra utrophin-deficiency produces an additional effect. Because of considerable variation in the data, however, the difference between mdx and mdx-utr did not reach statistical significance. In addition, to test for calcium-dependent inactivation, in several experiments, besides barium also calcium was used as charge carrier. Because of the comparably small calcium current amplitudes obtained (normally <150 pA), current decay was only analysed at one potential (0 mV), at which the elicited current was near maximum. The current decay kinetics (τ –values derived from single exponential decay fits) was 15±1 ms (n = 14) in wt, 23±2 ms (n = 13) in mdx, and 23±2 ms (n = 6) in mdx-utr. ANOVA revealed a significant difference (p<0.01) between the three tested groups. Tukey's Post Hoc test revealed significant differences both between mdx and wt (p<0.01), and between mdx-utr and wt (p<0.05), but not between mdx and mdx-utr. Together, these data suggest that calcium channel inactivation is significantly reduced in dystrophic neonatal cardiomyocytes. Finally, steady-state inactivation was similar in wt and dystrophic cells (Figure 2D).

### Sodium current properties in normal and dystrophic adult cardiomyocytes

**Figure 3:**
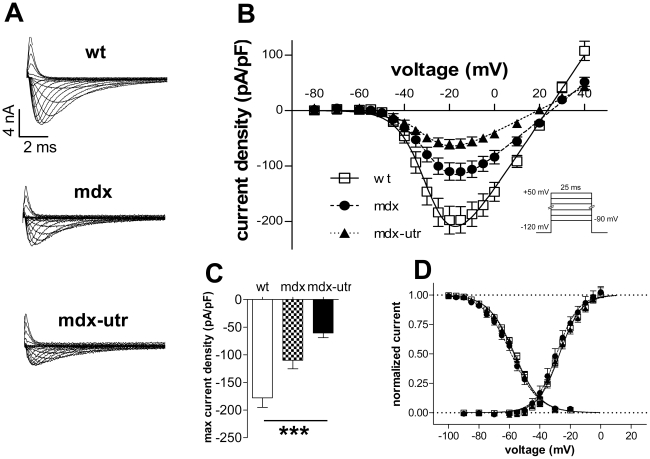
Sodium channel properties in wt and dystrophic adult cardiomyocytes. **A.** Original current traces of a typical wt, mdx, and mdx-utr cardiomyocyte, elicited by the pulse protocol in B. **B.** Current-voltage relationships derived from a series of experiments as shown in A. **C.** Maximum current densities in wt and dystrophic cardiomyocytes. *** indicates that ANOVA revealed a significant difference (p<0.001) between the three tested groups. **D.** Comparison of the voltage-dependencies of activation and steady-state inactivation between wt and dystrophic cardiomyocytes. For detailed statistics and n-values see Table 1.

To assess developmental changes in ion channel abnormalities in the dystrophic heart, we studied sodium channel properties also in cardiomyocytes derived from dystrophic adult (4–6 months old) mice. These were then compared with the respective findings on dystrophic neonatal cardiomyocytes. Figure 3 indicates that, as shown above for neonatal cardiomyocytes, the sodium current density was reduced in dystrophic compared to wt adult cardiomyocytes (B and C). Generally, this current density reduction was much more pronounced in dystrophic adult compared to dystrophic neonatal cardiomyocytes (compare Figures 1 and 3). In addition, in contrast to neonatal cells, in adult cardiomyocytes, extra utrophin-deficiency produced an additional effect on current density. Thus, we found a significant current density reduction in mdx-utr compared with mdx cardiomyocytes.

Figure 3D compares the sodium channel activation and steady-state inactivation between wt and dystrophic adult cardiomyocytes. These curves were similar in wt, mdx and mdx-utr cells. This is in contrast to the small but significant right shifts of both the activation and inactivation curves in neonatal mdx-utr cardiomyocytes, when compared to the wt and mdx (Figure 1D, Table 1).

### Action potentials in normal and dystrophic adult cardiomyocytes

**Figure 4:**
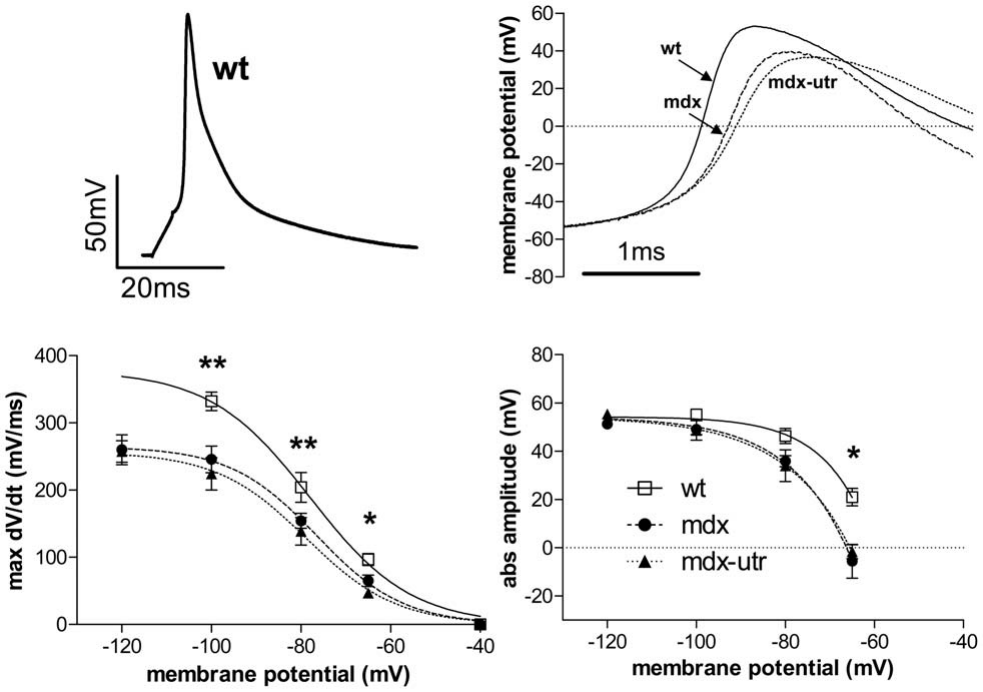
Action potential properties in wt and dystrophic adult cardiomyocytes. Typical AP of a wt cardiomyocyte (top left) elicited by a 4-ms current injection at 125% threshold. On the top right, the upstrokes of typical APs recorded from wt and dystrophic cardiomyocytes are overlayed on an expanded time scale. In the lower panel, maximum upstroke velocity (max. dV/dt), and the maximum depolarisation value reached by the AP (abs. amplitude) are plotted against the membrane potential. * and ** indicate significant differences (ANOVA; p<0.05 and <0.01; n-values: 5–8) between the three tested groups, respectively. Tukey's Post Hoc test revealed no significant differences between mdx-utr and mdx.

To estimate the effects of the strong sodium current reduction in dystrophic adult cardiomyocytes on the cardiac action potential (AP), we recorded APs from adult wt, mdx and mdx-utr cardiomyocytes (Figure 4). As expected, in these experiments, we found a significant decrease in AP upstroke velocity and amplitude in dystrophic cardiomyocytes (Figure 4, lower panel). A significant additional effect of extra utrophin-deficiency, as expected from the decreased sodium current density in mdx-utr compared to mdx cardiomyocytes, was not observed here. Such an effect was only existent by trend in the AP upstroke velocity. AP duration analyses at 30% repolarisation revealed 1.6±0.2 ms for wt, 2.1±0.5 ms for mdx, and 3.4±1.6 ms for mdx-utr. At 50% repolarisation, the respective values were 3.8±0.6 ms for wt, 4.1±1.2 ms for mdx, and 5.8±2.3 ms for mdx-utr. At 90% repolarisation AP durations were 24.2±4.2 ms in wt, 19±5.7 ms in mdx, and 25.1±9.9 ms in mdx-utr. No significant differences existed between any of these AP duration values. Finally, the resting membrane potential was −78±2 mV in wt, −76±3 mV in mdx, and −76±2 mV in mdx-utr.

### Surface electrocardiogram (ECG) recordings on normal and dystrophic neonatal and adult mice

**Figure 5:**
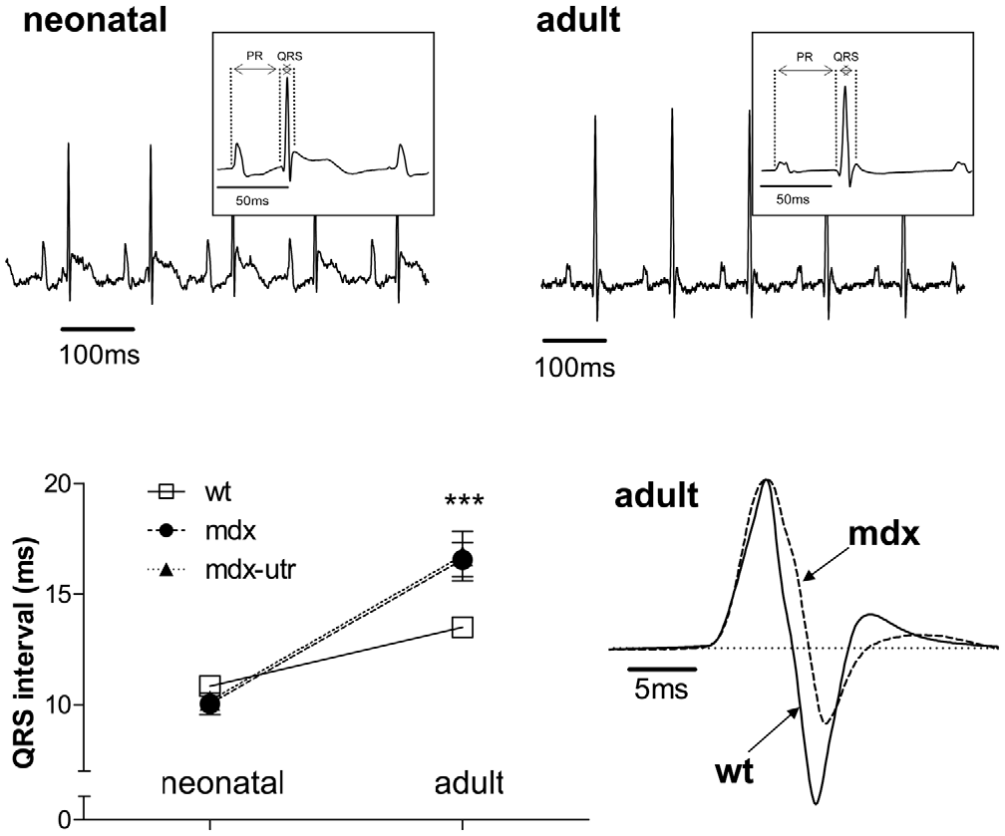
Surface ECG recordings on wt and dystrophic neonatal and adult mice. (Top) Typical original ECG signals (lead 1) from neonatal and adult wt mice, and the corresponding averages over 100 beats (insets). (Bottom right) Overlay of representative QRS-complexes (lead 2) in adult wt and mdx mice. In the graph, the QRS intervals of neonatal and adult ECGs are compared between wt and dystrophic mice. *** significant difference (ANOVA, p<0.001, n-values: 6–15) between the three tested groups. No significant differences exist between mdx-utr and mdx.

Reduced sodium currents and a slowed AP upstroke in dystrophic ventricular cardiomyocytes, as presented in this study, may affect cardiac ventricular conduction. This was tested by comparison of the QRS intervals derived from ECG recordings on wt and dystrophic mice. Figure 5 shows that, in neonatal mice, the QRS interval was very similar in wt and dystrophic animals. In adult mice, the QRS interval was significantly prolonged in dystrophic compared to wt animals, whereby extra utrophin-deficiency did not produce any additional effect (Figure 5). This suggests that the comparably moderate sodium channel impairments observed in dystrophic neonatal cardiomyocytes do not suffice to impair ventricular conduction. In contrast, the more severe sodium current reduction in dystrophic adult cardiomyocytes considerably slows ventricular conduction, independent of extra utrophin-deficiency. Further analyses of the ECG recordings shown in Figure 5 revealed PR interval durations of 30±1 ms for wt, 28±1 ms for mdx, and 27±1 ms for mdx-utr in neonatal mice. ANOVA revealed a significant difference (p<0.05) between the PR interval values of the three tested groups. Tukey's Post Hoc test for comparison between two groups revealed significant differences (p<0.05) between mdx and wt, and between mdx-utr and wt. In adult mice, the PR intervals were 49±1 ms in wt, 43±1 ms in mdx, and 43±2 ms in mdx-utr (p<0.01, ANNOVA). Tukey's Post Hoc test revealed significant differences (p<0.05) between mdx and wt, and between mdx-utr and wt. Finally the heart rates of neonatal mice were 624±15 beats per minute (bpm) in wt, 589±29 bpm in mdx, and 588±18 bpm in mdx-utr. In adult mice, the heart rates were 514±16 bpm in wt, 566±12 bpm in mdx, and 551±7 bpm in mdx-utr. Both in neonatal and adult mice, there was no significant difference between the heart rates of the three tested groups.

## Discussion

Here we report significant ion channel impairments in dystrophic neonatal and adult cardiomyocytes. Very recently, a study on the regulation of the Na_v_1.5 channel by utrophin in dystrophin-deficient mice was published [10], in which part of our results on adult cardiomyocytes were confirmed. In [10] adult cardiomyocytes derived from the same mouse models (mdx and mdx-utr), and animals of considerably younger age (8–10 weeks), compared with our study (4–6 months), were investigated. Similar to our results, [10] reported a significant reduction in sodium current density in mdx cardiomyocytes, and a further reduction in extra utrophin-deficient mdx-utr cardiomyocytes, which was accompanied by decreased Na_v_1.5 protein levels in the hearts of mdx-utr mice. In contrast to [10], we did not observe small but significant right shifts in the sodium current activation and steady-state inactivation curves in dystrophic adult cardiomyocytes. Interestingly, however, we found such shifts in extra utrophin-deficient neonatal cardiomyocytes (Figure 1D). The reason for this discrepancy between our findings and [10] is not known. It is, however, possible that the existing difference in animal age (see above) contributes, and that the positive shifts of the activation and inactivation curves disappear with older dystrophic animal age. A further discrepancy between our study and [10] lies in the additive affect of utrophin on the AP of mdx cardiomyocytes. Whereas [10] described a significant reduction of the AP upstroke velocity in mdx-utr compared with mdx cells, we did not find this significant difference, but only a minor trend in the same direction. In accordance with [10], on the other hand, we report similar AP amplitudes in mdx and mdx-utr cardiomyocytes.

### Ion channel dysfunction precedes cardiomyopathy development in the dystrophic heart

Heart failure leads to electrical remodelling including impairments of ion channel expression and function (reviewed in [24]). Since cardiomyopathy development and concomitant heart failure is also an important feature of the pathology observed in DMD patients [2], [3], as well as in DMD mouse models (e.g. [16]–[18]), it is likely that ion channel impairments also evolve in the dystrophic heart. All the studies which have previously reported aberrant sodium channel properties in dystrophic cardiomyocytes [5], [7], [10] were performed on adult mice. Thus, it is unclear, if the described sodium channel defects are primary effects of dystrophic gene mutations, or secondary effects resulting from the developing cardiac pathology. Importantly, here we show that significant sodium channel impairments are already present in dystrophic cardiomyocytes derived from neonatal mice. Since it is well established that cardiac abnormalities are absent in the used dystrophy mouse models even in young adult animals (e.g. [16], [18], [21]–[23]), these sodium channel defects in dystrophic neonatal cardiomyocytes can be considered primary effects of the dystrophic gene mutations, which precede cardiomyopathy development. Our additional finding of a significantly reduced calcium channel inactivation in dystrophic neonatal cardiomyocytes suggests that this is not a special feature of sodium channels alone, but more generally applies to other voltage-gated ion channels as well.

Recently, transgenic mice with reduced expression of Na_v_1.5 channels in the heart were shown to develop a dilated cardiomyopathy [13]. This work, together with a study on heterozygous SCN5A-knockout mice [25], and reports associating mutations in the SCN5A gene with cardiomyopathy (e.g. [14], [15]), suggests that aberrant Na_v_1.5 channel expression and function are underlying mechanisms for cardiomyopathy. Since we found both reduced sodium currents (mdx, mdx-utr) and impaired sodium channel gating (only mdx-utr) in dystrophic neonatal cardiomyocytes, this may also be true for dystrophic cardiomyopathy. In this context it is tempting to speculate that the additional sodium channel gating impairments, only present in neonatal mdx-utr- but not in mdx- cardiomyocytes, may contribute to the more severe cardiomyopathy development observed in mdx-utr compared with mdx mice [16], [17], [20]. Indeed, these gating impairments may even represent the crucial cardiomyopathy worsening factor, when considering that the sodium current densities in neonatal mdx and mdx-utr cardiomyocytes were similar.

Besides reduced sodium currents in cardiomyocytes [13], also increased calcium currents may initiate cardiomyopathy development. Thus, transgenic mice with an increased number of L-type calcium channels in the heart developed a severe cardiomyopathy [26]. Here we report considerably reduced calcium channel inactivation in dystrophic neonatal cardiomyocytes, which should enhance calcium influx through these channels. This may lead to increases in intracellular calcium, and in line with [26], possibly also contribute to cardiomyopathy development.

Finally, our finding of a more severe sodium current reduction in dystrophic adult compared to dystrophic neonatal cardiomyocytes may suggest that the developing pathology in the dystrophic heart also generates secondary effects on the sodium channel properties. If so, the stronger sodium current reduction we found in adult mdx-utr, compared with adult mdx cardiomyocytes, may be related to the more severe cardiomyopathy observed in mdx-utr mice. Thus, whereas in mdx mice a cardiomyopathy evolves comparably slow (e.g. [18]), in the mdx-utr mouse model, cardiac abnormalities including cardiomyocyte necrosis, inflammation, fibrosis and contractile dysfunction are known to be strongly present in the animal age range used in this study (4–6 months) [16], [17], [20].

### Extra utrophin-deficiency worsens electrophysiological abnormalities in mdx cardiomyocytes

We show here that extra utrophin-deficiency generates significant sodium current abnormalities in addition to those produced by dystrophin-deficiency only. Similar results were recently reported by [10]. Whereas [10] studied cardiomyocytes from young adult (8–10 weeks) dystrophic (mdx and mdx-utr) mice only, we have investigated cardiomyocytes from neonatal and considerably older adult (4–6 months) dystrophic mice, thereby providing new information on the development and progression of the sodium channel impairments in extra utrophin-deficient dystrophic cardiomyocytes. Our findings are in accordance with a strong regulatory effect of utrophin on cardiac Na_v_1.5 channels in dystrophin-deficient mdx mice. This may occur by indirect interaction of utrophin with Na_v_1.5 via syntrophins [10].

A recent study [27] suggests that, besides improving Na_v_1.5 channel impairments in the case of dystrophin-deficiency, utrophin may also rescue other impaired electrophysiological properties. Thus, these authors reported a more severe impairment in skeletal muscle excitation-contraction coupling in mdx-utr compared to mdx mice. Since utrophin interacts with several protein members of the DAPC [28], [29], and DAPC members interact with ion channels (e.g. [7], [30]), the described rescue effects by utrophin may be achieved by restoring the integrity of the disrupted DAPC when dystrophin is absent.

### Sodium channel impairments in adult cardiomyocytes affect conduction in the dystrophic heart

Since the sodium channel impairments in dystrophic neonatal and adult ventricular cardiomyocytes are considerably different, namely more severe in adult cells, it was of interest to test their functional relevance, by comparing their impacts on cardiac ventricular conduction. The results suggest that the sodium channel impairments in dystrophic neonatal cardiomyocytes do not suffice to delay ventricular conduction. This is suggested by similar QRS intervals in ECG recordings on wt and dystrophic neonatal mice. Concomitant with a pronounced retardation of the AP upstroke in dystrophic adult cardiomyocytes, ECG recordings on adult mice, in contrast to those on neonatal ones, revealed considerably prolonged QRS intervals in dystrophic compared to wt animals. In line with previous mdx mouse studies [5], [7], [31], this suggests that ventricular conduction is considerably slowed in dystrophic adult animals, most probably due to the strongly reduced sodium channel availability. An additional QRS interval prolongation by extra utrophin-deficiency in adult animals, as would be expected from the pronounced sodium current reduction especially in mdx-utr cardiomyocytes, was not observed here. In contrast, QRS was very similar in adult mdx and mdx-utr mice, and thus, independent of extra utrophin-deficiency. The reason for this surprising finding is not known. It may be explained by alterations in other factors than sodium channels, which also modulate cardiac conduction, and counteract the effects generated by reduced sodium currents.

Finally, in contrast to similar QRS intervals in neonatal dystrophic and wt mice, the PR interval of the ECG (reflecting AV nodal conduction) was significantly shortened already in dystrophic neonatal animals. This may be related to enhanced calcium currents due to reduced calcium channel inactivation in dystrophic neonatal cardiomyocytes. This hypothesis, however, requires that calcium channel inactivation is similarly impaired in AV nodal cardiomyocytes as in ventricular cardiomyocytes, which was not verified in this study. The PR interval shortening, which was independent of extra utrophin-deficiency, was more pronounced in dystrophic adult mice, and matches with previous studies (e.g. [31], [32]).

### Clinical implications

If the dystrophin/utrophin double mutant mouse is indeed a suitable model for DMD with representative cardiac pathology (probably superior to the mdx mouse), as suggested by several groups (e.g. [16], [19], [20]), our finding of a more severe cardiac sodium channel dysfunction in this mouse model has an important implication: sodium channel impairments may be considered a major cause of the cardiac conduction defects observed in DMD patients [11], [12], probably as yet underestimated when using the common mdx mouse as model system. This critical loss of sodium current in dystrophic cardiomyocytes may be further corroborated by increasing myocardial fibrosis during disease progression. Thus, paracrine factors released from cardiac fibroblasts slow impulse conduction and reduce Na_v_1.5 channel expression in cardiomyocytes [33]. Consequently, drugs that directly enhance cardiac sodium currents, or strategies indirectly enhancing sodium currents, e.g. by overexpression of endogenous utrophin, may be useful to restore normal conduction and prevent life-threatening arrhythmias in DMD patients.

A second important implication of our study emerges from the finding that ion channel impairments precede cardiomyopathy development in the dystrophic heart. Both reduced sodium currents [13] and increased calcium currents [26] in cardiomyocytes are considered potential underlying mechanisms for cardiomyopathy development. Since the ion channel impairments we found in dystrophic neonatal cardiomyocytes prior to cardiac pathology development, entail exactly these effects on the named currents, we speculate that they contribute to initiate cardiomyopathy development in the dystrophic heart. If so, drugs that enhance sodium currents and/or decrease calcium currents can be considered strategies to prevent dystrophic cardiomyopathy development.

## Materials and Methods

The study coincides with the rules of the University Animal Welfare Committee, and conforms with the European Commission Directive 86/609/EEC. For animal research, approval was granted by the Austrian Ministry for Science and Research (BMWF-66.009/0211-II/10b/2009).

### Mouse models

Normal wild type (wt) C57/BL6 mice, dystrophin-deficient mdx [9] and dystrophin/utrophin-deficient double mutant mdx-utr [16], [19] mice, backcrossed on the C57/BL6 background for more than 10 generations, were used for the study. Throughout the text, the dystrophin−/− status (utrophin+/+) is termed “mdx”. Double mutant (dystrophin−/− and utrophin−/−) mice are referred to as “mdx-utr”. The mice were genotyped by standard PCR-assays. For comparisons between mdx and mdx-utr, littermates were used in all electrophysiological experiments.

### Preparation of neonatal ventricular cardiomyocytes

Cardiomyocytes were isolated from the ventricles of neonatal (1–2 days) mouse hearts. After decapitation of the mice, hearts were rapidly excised, and the ventricular tissue was mechanically dissociated, followed by proteolytic digestion. The isolated cardiomyocytes were grown on Matrigel-coated (Becton Dickinson, Schwechat, Austria) culture dishes. A detailed description of the procedures for the isolation and culture of neonatal cardiomyocytes is given in our earlier work [34].

### Isolation of adult ventricular cardiomyocytes

Ventricular cardiomyocytes were isolated from female mice, 4–6 months of age, by using a Langendorff setup, which was custom-built in our lab. Animals were killed by cervical dislocation, and 4 ml ice-cold Ca^2+^-free solution (see below) was injected in the ventricles to stop contractions and rinse free of blood. Hearts were rapidly excised and a cannula was inserted into the aorta for retrograde perfusion with Ca^2+^-free solution (in mM: 134 NaCl, 11 glucose, 4 KCl, 1.2 MgSO_4_, 1.2 Na_2_HPO_4_, 10 Hepes, pH adjusted to 7.35 with NaOH) containing 0.17 mg/ml Liberase^TH^ (Roche) at 37°C for 18 min. To increase the viability of the cardiomyocytes, 10 mM 2, 3-butanedione monoxime (BDM, Sigma) was also added. The ventricles were then cut into pieces, incubated on a shaker at 37°C, and calcium concentration was increased to 200 µM over one hour in five steps. Pieces of digested ventricular tissue were then triturated to liberate cardiomyocytes. After centrifugation (3 min, 500 rpm), cells were resuspended in Minimum Essential Medium (MEM) alpha (Gibco), containing ITS media supplement (Sigma) diluted 1∶100 (final concentration of 10 µg/ml insulin, 5.5 µg/ml transferrin, 5 ng/ml selenite), 4 mM L-glutamine, 50 u/ml penicillin, 50 µg/ml streptomycin and 25 µM blebbistatin (Sigma). Cells were plated on Matrigel (Becton Dickinson)-coated culture dishes.

### Electrophysiological studies using the whole cell patch clamp technique

A detailed description of the electrophysiological recordings is given in our earlier work [34]. Ionic currents were recorded from neonatal cardiomyocytes up to 24 hours, and from adult cardiomyocytes up to 8 hours after preparation at room temperature (22±1.5°C), using an Axoclamp 200B patch clamp amplifier (Axon Instruments, Union City, CA). Pipettes were formed from aluminosilicate glass (AF150-100-10; Science Products, Hofheim, Germany) with a P-97 horizontal puller (Sutter Instruments, Novato, CA), and had resistances between 0.8 and 2 MΩ when filled with the respective pipette solutions (see below). Data acquisition was performed with pClamp 6.0 software (Axon Instruments) through a 12-bit A-D/D-A interface (Digidata 1200; Axon Instruments). Data were low-pass filtered with 1–10 kHz (−3 dB) and digitized at 10–100 kHz. Data analysis was performed using Clampfit 10.2 (Axon Instruments) and GraphPad Prism 5.01 (San Diego, USA) software.

#### Sodium currents

Current-voltage (IV) relationships were fit with the function: I = G_max_*(V−V_rev_)/(1+exp((V_0.5_−V)/K)) where I is the current, G_max_ is the maximum conductance, V is the membrane potential, V_rev_ is the reversal potential, V_0.5_ is the voltage at which half-maximum activation occurred, and K is the slope factor. Decay half-time, a measure of fast inactivation kinetics, was obtained by analysing the time period between the current peak and the time point at which the current had decayed to 50%. Steady-state fast inactivation data were fit with the Boltzmann function: I/I_max_ = 1/(1+exp((V−V_0.5_)/K)) where I/I_max_ is the normalized current, V is the membrane potential, V_0.5_ is the voltage at which half-maximum inactivation occurred, and K is the slope factor. Recordings were made in a bath solution that consisted of 15 mM NaCl, 125 mM N-methyl-D-glucamine (NMDG), 2.5 mM KCl, 1 mM CaCl_2_, 1 mM MgCl_2_, 10 mM HEPES, pH = 7.4 adjusted with HCl. The pipette solution contained 105 mM CsF, 10 mM NaCl, 10 mM EGTA, 10 mM HEPES, pH = 7.3 adjusted with CsOH. Chemicals were purchased from Sigma. Rapid solution changes were performed by a DAD-8-VC superfusion system (ALA Scientific Instruments, Westbury, NY).

#### Barium currents

The external solution contained 10 mM BaCl_2_, 145 mM TEA-Cl, 10 mM Hepes, pH = 7.4 adjusted with TEA-OH. The internal solution contained 145 mM Cs-aspartate, 2 mM MgCl_2_, 10 mM Hepes, 0.1 mM Cs-EGTA, 2 mM Mg-ATP, pH = 7.4 adjusted with CsOH. The kinetics of barium current inactivation was analysed by fitting the current decay after channel activation with a single exponential function. In experiments where calcium was used as charge carrier, the external solution contained 10 mM CaCl_2_ instead of 10 mM BaCl_2_.

#### Action potential recordings

APs were recorded from adult cardiomyocytes derived from 4–6 months old female mice in the current-clamp mode of the whole cell patch clamp technique. APs were elicited at 1 Hz from defined membrane potentials (fixed by continuous current injection) by rectangular current pulses of 4 ms duration at 125% threshold level. The cells were bathed in 140 mM NaCl, 4 mM KCl, 2 mM CaCl_2_, 2 mM MgCl_2_, 5 mM Hepes, 5 mM Glucose, pH = 7.4 adjusted with NaOH. The pipette solution contained 10 mM NaCl, 140 mM KCl, 2 mM EGTA, 1 mM MgCl_2_, 0.1 mM Na-GTP, 5 mM Mg-ATP, 10 mM Hepes, pH = 7.2 adjusted with KOH.

### Surface electrocardiogram recordings

Standard 6-lead surface electrocardiograms (ECGs) were recorded from conscious neonatal (8–14 days) and anaesthetized adult (4–6 months) mice. ECG recordings from neonatal mice were performed as in [35]. In brief, a custom-built setup restrained neonatal mice in movement. Four Ag/AgCl-electrode footpads were used to record ECGs. The pads were wetted with ECG electrode gel, and each foot was placed such that it made contact with a separate electrode. Adult mice were anaesthetized with 350 mg/kg chloral hydrate (single injection, ip), and depth of anaesthesia was monitored (minimal response to hind-foot pinch). Small-needle ECG leads were placed subcutaneously on all 4 extremities. Body temperature was maintained at 37°C using a heating pad and lamp. Signals were amplified (Gould model 11 G412301; Gould Inc., Cleveland, Ohio, USA), high- and low-pass filtered with 3 dB cut-off frequencies of 0.3 and 1 kHz, respectively, digitized at 5 kHz, and stored for offline analysis. To reduce noise levels, ECG signals were averaged over 100 beats prior to evaluation. QRS interval values were determined by calculating the mean of all 6 standard leads. The duration of the QRS interval was measured from the sharp onset to the offset of depolarization as in [36]. The PR interval was measured from the beginning of the P wave to the beginning of the QRS complex (see insets in Figure 5).

Data are expressed as means ± SEM. Statistical comparisons were made using one way ANOVA (for independent samples) and Tukey's Post Hoc test. A p-value<0.05 was considered significant.
